# The Effect of Biotinylated PAMAM G3 Dendrimers Conjugated with COX-2 Inhibitor (celecoxib) and PPARγ Agonist (Fmoc-L-Leucine) on Human Normal Fibroblasts, Immortalized Keratinocytes and Glioma Cells in Vitro

**DOI:** 10.3390/molecules24203801

**Published:** 2019-10-22

**Authors:** Łukasz Uram, Maria Misiorek, Monika Pichla, Aleksandra Filipowicz-Rachwał, Joanna Markowicz, Stanisław Wołowiec, Elżbieta Wałajtys-Rode

**Affiliations:** 1 Faculty of Chemistry, Rzeszów University of Technology, 6 Powstańców Warszawy Ave, 35-959 Rzeszów, Poland; mczygier@prz.edu.pl (M.M.); j.markowicz@outlook.com (J.M.); 2 Department of Cosmetics and Pharmaceutical Products Technology, Rzeszów University of Information Technology and Management, 2 Sucharskiego Str, 35-225 Rzeszów, Poland; afilipowicz@poczta.wsiz.rzeszow.pl; 3 Faculty of Medicine, University of Rzeszów, Warzywna 1a, 35-310 Rzeszow, Poland; swolowiec@ur.edu.pl; 4 Department of Drug Technology and Biotechnology, Faculty of Chemistry, Warsaw University of Technology,75 Koszykowa Str, 00-664 Warsaw, Poland; ewalajtys@ch.pw.edu.pl

**Keywords:** biotinylated PAMAM G3 dendrimer, COX-2 inhibitor—celecoxib, PPARγ agonist—Fmoc-L-Leucine, human fibroblast, glioblastoma and immortalized keratinocytes

## Abstract

Glioblastoma multiforme (GBM) is the most malignant type of central nervous system tumor that is resistant to all currently used forms of therapy. Thus, more effective GBM treatment strategies are being investigated, including combined therapies with drugs that may cross the blood brain barrier (BBB). Another important issue considers the decrease of deleterious side effects of therapy. It has been shown that nanocarrier conjugates with biotin can penetrate BBB. In this study, biotinylated PAMAM G3 dendrimers substituted with the recognized anticancer agents cyclooxygenase-2 (COX-2) inhibitor celecoxib and peroxisome proliferator-activated receptor γ (PPARγ) agonist Fmoc-L-Leucine (G3-BCL) were tested in vitro on human cell lines with different p53 status: glioblastoma (U-118 MG), normal fibroblasts (BJ) and immortalized keratinocytes (HaCaT). G3-BCL penetrated efficiently into the lysosomal and mitochondrial compartments of U-118 MG cells and induced death of U-118 MG cells via apoptosis and inhibited proliferation and migration at low IC_50_ = 1.25 µM concentration, considerably lower than either drug applied alone. Comparison of the effects of G3-BCL on expression of COX-2 and PPARγ protein and PGE_2_ production of three different investigated cell line phenotypes revealed that the anti-glioma effect of the conjugate was realized by other mechanisms other than influencing PPAR-γ expression and regardless of p53 cell status, it was dependent on COX-2 protein level and high PGE_2_ production. Similar G3-BCL cytotoxicity was seen in normal fibroblasts (IC_50_ = 1.29 µM) and higher resistance in HaCaT cells (IC_50_ = 4.49 µM). Thus, G3-BCL might be a good candidate for the targeted, local glioma therapy with limited site effects.

## 1. Introduction

Glioblastoma multiforme (GBM), classified by WHO as grade IV, is the most lethal primary tumor of the central nervous system (CNS), with high prevalence in developed, industrialized countries [1,2]. The particularly invasive nature of GBM is the main source of recurrence [3]. The present, conventional GBM treatment that includes surgery combined with radiotherapy and chemotherapy with temozolomide (TMZ) has limited clinical efficacy and patient prognosis remains poor with a survival rate of 15 months after diagnosis and 5-year survival of 5% of patients [4,5,6,7].

Among major signaling pathways affected in GBM, the mutation/inactivation of gene p53, a main switch coordinating cell fate between survival and death has been found in 54–87% of high-grade human gliomas [8,9,10]. The p53 mutations are associated with short survival time and resistance to chemo and radio therapy and the same mutants are known to gain oncogenic functions and promote tumorigenesis and cancer progression [11,12]. Thus, it is a matter of importance to recognize of the effectiveness of applied therapeutics against cancer cells with modified p53 status as compared with normal or non-cancerogenic immortalized cells with mutated p53.

In the search for effective GBM therapy, new approaches are considered (for a review see [13]). Among them, combined therapies using the small molecule inhibitors of cell signaling pathways that are overexpressed in gliomas are under investigation [14]. Prominent signaling pathways have been recognized that control tumor development, progression and malignancy. The canonical Wnt/β-catenin signaling pathway, the COX-2 and peroxisome proliferator-activated receptor gamma (PPARγ) activities that operate in opposing manner have an impact on regulation of tumor growth, including GBM. It was revealed that in many cancers including gliomas, the Wnt/β-catenin pathway and COX-2 expression are upregulated while PPARγ is downregulated [15,16,17,18]. The interaction between these pathways is complicated and elicited by various molecular mechanisms. Under normal homeostasis, PPARγ agonists directly inhibit activation of β-catenin and transcription of Wnt/β-catenin targeted genes, including COX-2, that stop cancer development and progression [19]. During cancerogenesis, the overexpression of COX-2 and high level of its metabolite prostaglandin E2 (PGE_2_) in GBM cells correlate with a substantial increase of the rate of cell proliferation, adhesion, migration, angiogenesis and metastasis, as well as with immunosuppression of defense mechanisms [20,21,22,23]. However, recent data has revealed that there is no simple relation between COX-2 activity and PGE_2_ level and antitumor action of their inhibitors, due to diverse effects of compounds on cellular metabolism and various signaling pathways [24,25,26].

Among nonsteroidal anti-inflammatory drugs (NSAIDs) that affect the Wnt/β-catenin pathway, celecoxib inhibits β-catenin signaling by cyclooxygenase (COX-2)-dependent and COX-2-independent mechanisms and has demonstrated anticancer activity [27,28]. COX-2 inhibition decreases cellular levels of fatty acids and their derivatives supplied by the lipooxygenase and cyclooxygenase pathways that are PPAR-γ ligands and therefore indirectly affect PPAR-γ activity [29,30]. On the other hand, COX-2 inhibitors and certain prostaglandins (15d-PGJ2) bind and activate the nuclear receptor PPARγ [31]. It has been also shown that anti-tumor mechanisms of the COX 2 inhibition might be either p53-dependent or p53-independent in various cancerous and non-cancerous cells. In glioma cell lines with active p53, celecoxib effectively inhibits viability and proliferation whereas in lines with inhibited or mutated p53, no induction of apoptosis is observed [32].

Overexpression of PPARγ is also a hallmark of GBM cells [33] and it has been documented that the PPARγ agonists thiazolidinediones and Fmoc-L-Leucine inhibit growth, proliferation, and induce apoptosis in various human glioblastoma cells in vivo, and thus may be regarded as a potential agents for GBM therapy [34,35,36,37,38,39]. The obvious conclusion is to combine both factors to potentiate anti-glioma effects [31]. So far, the synergistic anti-cancer action of these two factors was shown in in vivo and in vitro studies [40,41,42]. Also, our earlier study showed additive effects of celecoxib (COX-2 inhibitor) and Fmoc-L-Leucine (PPARγ agonist) conjugated with biotinylated PAMAM G3 dendrimer in glioma and squamous cell carcinoma in vitro [39]. However, a limitation of the described therapy is its toxic side effects. It is known that dependent and independent pro-proliferative effects receptor PPARγ agonists lead to increased risk of cardiovascular disease and peripheral fractures [43,44,45]. Also, COX-2 inhibitor treatment is associated with an increased risk of adverse cardiovascular events [46]. So, lowering the therapeutic concentrations of described drugs also becomes an important issue [47,48].

Particular attention has been given to poly(amidoamine) dendrimers (PAMAM) as drug carriers, due to their beneficial physico-chemical properties and reasonable biocompatibility [49,50]. These cationic nanocarriers bearing hydrophilic surfaces have been shown to be suitable for delivering drugs to brain tumors [51,52]. The penetration of the blood brain barrier (BBB) by dendrimers and accumulation in malignant glioma was demonstrated for functionalized dendrimers [53]. Positive results were obtained with PAMAM dendrimers in glioma in vivo imaging [54] and in various types of drug delivery into gliomas [55]. Biotin is considered as a particularly promising ligand that can transfer conjugated drugs across the BBB and target brain tumors [56,57]. Biotin binds to specific surface receptors (Sodium Dependent Multivitamin Transporter, SMVT) or monocarboxylate transporters (MCT-1 and MCT-8) overexpressed in a majority of cancer cells [58,59,60]. Veszelka et al. (2017) showed that biotin increased transfer of solid nanoparticles (SNP) across brain endothelial hCMEC/D3 cells (BBB model) with expression of mRNA for SMVT (SMVT/SLC5A6) gene. After 8 h of incubation, uptake of the biotin-SNP was two times higher than the uptake of SNP alone. Also, the permeability of BBB upon biotin-SNP treatment was 2.8-fold higher than that of SNP. Moreover, biotinylated glutathione was shown to increase nanoparticle permeability through endothelial monolayers supporting its use as a brain targeting vector [61]. Also, our earlier studies revealed the usefulness of biotinylated PAMAM G3 dendrimers as a vehicle of perspective anti-glioma drugs (celecoxib and Fmoc-L-Leucine). Biotinylated PAMAM G3 dendrimers substituted with those drugs revealed higher cytotoxicity against U-118 MG glioma cells as compared to single drugs and were delivered more efficiently than non-biotinylated constructs [39,62].

The major aim of this study was to gain more information concerning the possibility of obtaining a nanocarrier with both COX-2 inhibitor and PPARγ agonist, for use as a potential local GBM therapeutic. For this purpose, PAMAM G3 dendrimer was conjugated with 16 celecoxib molecules (COX-2 inhibitor), 15 molecules of Fmoc-L-Leucine (agonist of PPARγ) and one tumor-targeting biotin molecule (G3-BCL) [39]. To evaluate the cytotoxic effect of the G3-BCL conjugate, three human cell lines with various phenotypes were chosen. Glioblastoma U-118 MG classified as grade IV GBM and immortalized keratinocytes (HaCaT) have confirmed mutations in the p53 gene that generally are characteristic for development of cancer. However, the immortalized HaCaT line is not cancerogenic. For comparison, normal fibroblasts (BJ) with active p53 were also included. The biological properties of G3-BCL conjugate were assessed by determining viability, mechanisms of cell death (apoptosis/late apoptosis-necrosis) and mobility, cellular energy level (ATP), expression of COX-2 and PPAR γ proteins and PGE_2_ production. Intracellular accumulation and localization of a fluorescent labeled G3-BCL analogue were also performed. The results obtained showed that G3-BCL conjugate revealed much higher cytotoxicity and inhibition of mobility of the U-118 MG cells (at 1-4 µM concentration) as compared to either drug applied alone. HaCaT cells exhibited higher resistance to G3-BCL conjugates. The interesting results concern the high mitochondrial localization of investigated conjugate in U-118 MG cells and induction of cell death preferentially by an apoptotic mechanism. Results showed a similar sensitivity of normal fibroblasts to conjugate treatment that limits the application of obtained nanocarrier to localized treatment.

## 2. Results and Discussion

### 2.1. Cytotoxicity of G3-BCL Conjugate

**Figure 1:**
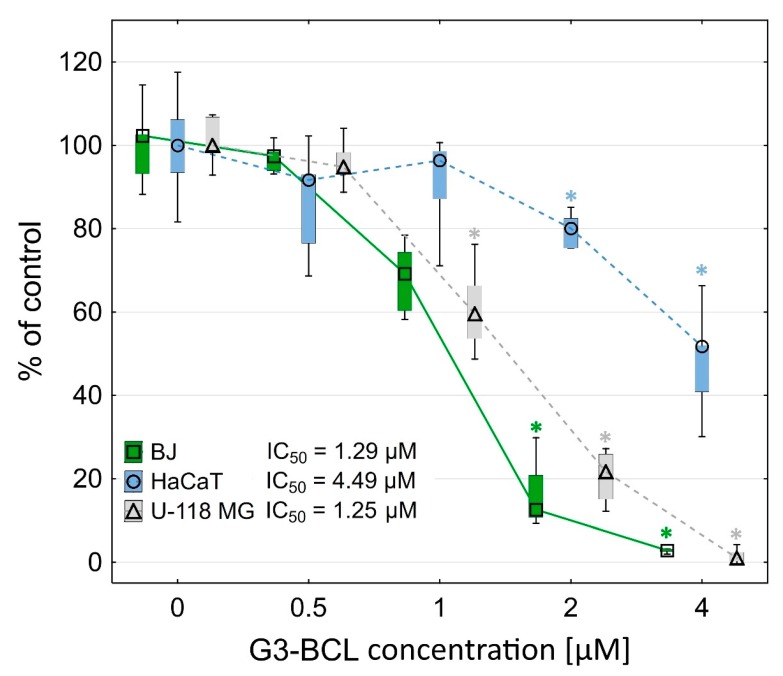
Cytotoxicity of G3-BCL conjugate for BJ, HaCaT and U-118 MG cells after 24 h treatment, estimated by XTT assay. Results are presented as median of triplicate assays from three independent experiments and expressed as % of non-treated controls. The whiskers are lower (25%) and upper (75%) quartile ranges. * P < 0.05; Kruskal-Wallis test.

The G3-BCL construct was toxic at a low concentration range for glioma cells (IC_50_ = 1.25 µM) and normal human fibroblasts (IC_50_ = 1.29 µM), whereas immortalized keratinocytes were more resistant (IC_50_ = 4.49 µM) (Figure 1).

### 2.2. Proliferation

**Figure 2:**
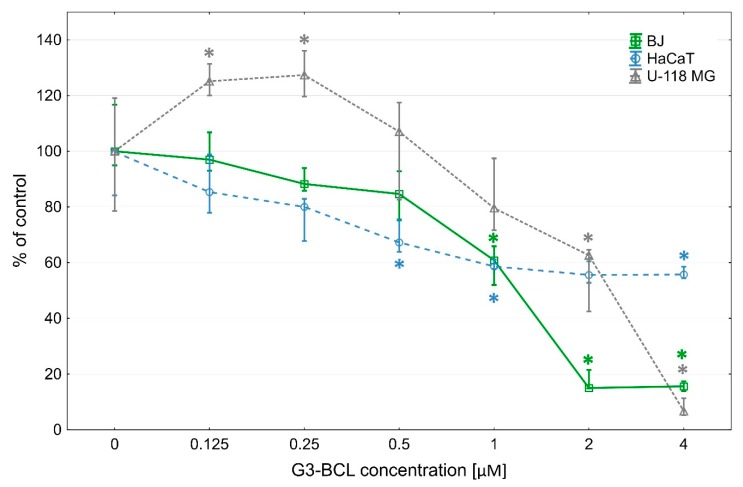
Proliferation of investigated cells after 72 h incubation with G3-BCL conjugate. Results are presented as median of triplicate from three independent experiments and expressed as % of non-treated controls. The whiskers are lower (25%) and upper (75%) quartile ranges. * P < 0.05; Kruskal–Wallis test.

Inhibition of proliferation of the fibroblasts by G3-BCL conjugate was observed at a range of IC_50_ concentration similar to its cytotoxicity. However, in U-118 MG cells, a two-phase effect was seen. At low, non-toxic concentrations (up to 0.25 µM), proliferation rate increased to 120% of control and significant decrease was visible at IC_50_ concentration (60% of control). In HaCaT cells, decrease of proliferation rate begun at 0.5 µM concentration and at IC_50_ concentration amounted to 60% of control (Figure 2).

Celecoxib and parecoxib, the prodrug of valdecoxib from the same group as celecoxib, significantly reduced the proliferation of a variety of GBM cell lines, however, at much higher concentrations of 50–100 µM and 100–200 µM, respectively [63,64].

### 2.3. Cellular Accumulation of G3-BCLF

**Figure 3:**
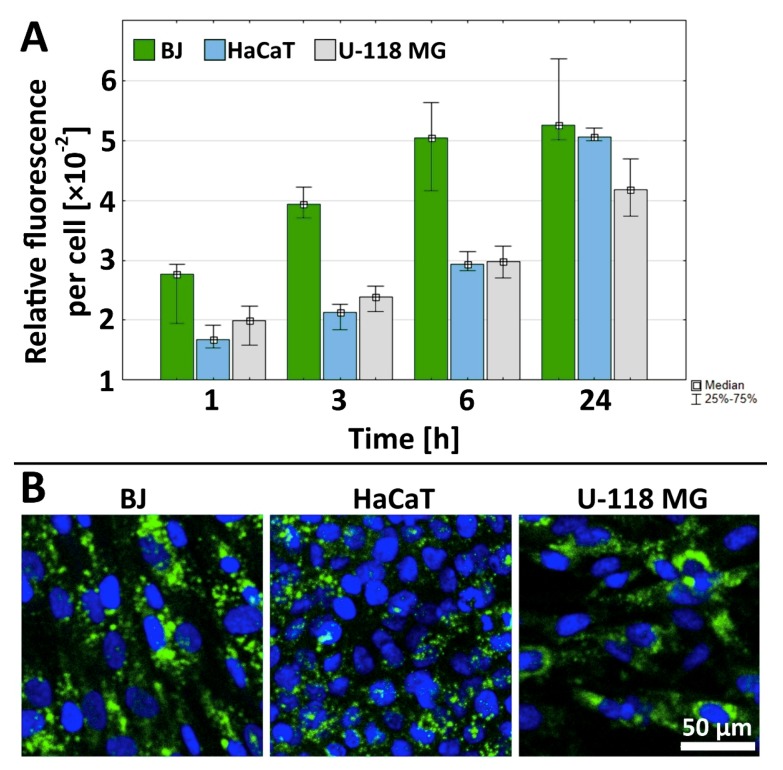
(**A**) Time–dependent cellular accumulation of 0.1 µM G3-BCLF conjugate in BJ, HaCaT and U-118 MG cells. Results are presented as median of triplicate assays from three independent experiments and expressed as a fluorescence signal (relative fluorescence units) per cell. The whiskers are lower (25%) and upper (75%) quartile ranges. (**B**) Representative images of the accumulation of G3-BCLF conjugate after 24 h incubation (green signal) and DAPI labeled nuclei (blue signal).

Time-dependent uptake of 0.1 µM G3-BCLF by fibroblasts reached a steady state after 6 h, whereas both HaCaT and glioma cells accumulated the conjugate in a concentration-dependent manner up to 24 h; however, this was at a lower level than fibroblasts (Figure 3A and confocal imaging Figure 3B). This observation points to more efficient biotin-conjugate transport into normal fibroblasts as compared to cancer U-118 MG cells and immortalized keratinocytes. This may be due to the described phagocytic activity of fibroblasts [65]. The lower accumulation of G3-BCLF in U-118 MG cells may be the result of the active xenobiotic efflux systems present in cancer cells since our earlier studies revealed similar fluorescent labeled biotin accumulation in BJ, HaCaT and U-118 MG cells assayed under the same conditions [39]. In HaCaT cells, the high detoxifying II phase enzymes activity was found that may be responsible for the observed lower level of G3-BCLF accumulation as compared to fibroblasts [66].

### 2.4. Subcellular Localization of G3-BCLF

**Figure 4:**
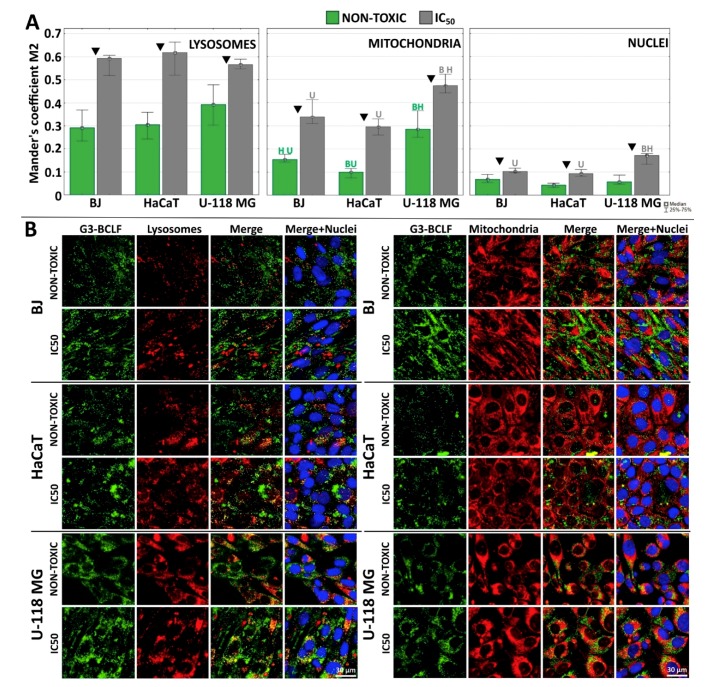
(**A**) Localization of G3-BCLF in lysosomes, mitochondria and nuclei of BJ, HaCaT and U-118 MG cells after 24 h incubation at low, non-toxic (0.1 µM) and equal to IC_50_ concentrations. Results are medians of twelve images measurement from each experimental group and expressed as Mander’s coefficient. The whiskers are lower (25%) and upper (75%) quartile ranges. Letters indicate significant differences between cell lines (each letter indicate cell line), P < 0.05; Kruskal-Wallis test. ▼ P < 0.05; Mann–Whitney U-test (non-toxic against IC_50_ concentration). (**B**) Confocal images presenting colocalization of G3-BCLF within lysosomes, mitochondria and nuclei in investigated cell lines. Green signal: G3-BCLF, red: lysosomes (left panel) or mitochondria (right panel), blue: DAPI stained nuclei, yellow: overlapping signal from G3-BCLF and lysosomes or mitochondria. Scale bar equal 30 µm.

The subcellular localization of fluorescent G3-BCLF was measured at low, nontoxic (0.1 µM) and concentrations equal to IC_50_ (IC_50_ = 1.25, 1.29, 4.49 µM for U-118 MG, BJ and HaCaT, respectively). The estimation of Mander’s coefficient (M2) of the G3-BCLF in all investigated cell lines indicated following colocalization efficiency after 24 h treatment: lysosomes > mitochondria > nuclei (Figure 4A,B). The M2 coefficient for lysosomal localization revealed similar and a rather high value > 0.6 at IC_50_ concentrations for all investigated cell lines. Studies with confocal laser scanning microscopy revealed that unmodified and surface modified G3 PAMAM dendrimers are internalized and trafficked to endosomes and lysosomes [67]. High levels of G3-BCLF in lysosomes may indicate active macropinocytosis or clatrin-mediated endocytic transport of conjugate [68]. Also, Albertazzi et al. showed that cationic and neutral PAMAM dendrimers are internalized through clathrin-dependent endocytosis and macropinocytosis, with cargo redirected into the lysosomes (high degree of colocalization coefficient) [69]. Kitchens et al. indicated that PAMAM dendrimer internalization was realized via endocytosis pathway with vesicular trafficking and clathrin-dependent endocytosis [70,71]. The G3-BCL dendrimer in this study might also be internalized partially by biotin receptor-mediated endocytosis or charge-mediated adsorptive endocytosis. Yellepeddi et al. showed that in ovarian cancer (OVCAR-3) and human embryonic kidney (HEK 293T) cells cellular uptake of biotin-PAMAM G4 dendrimers occurred as described above [72]. It is also possible that celecoxib and Fmoc-L-Leucine residues attached to the surface of biotinylated PAMAM molecule have an impact on the internalization pathway and distribution of the tested compound in the cell. However, this issue requires further and more detailed research.

The nuclear envelope proved to be a barrier for the conjugate. At non-toxic concentrations of G3-BCLF, Mander’s coefficient did not exceed the value 0.07 in all cell lines and at IC_50_ concentrations, the M2 value was < 0.2 in glioma and < 0.1 in BJ and HaCaT cells (Figure 4A). This is in good agreement with data obtained by the others concerning cellular biotin localization [73]. Most interesting is a high localization of G3-BCLF in mitochondria of U-118 MG cells. M2 at non-cytotoxic concentration was 0.28 and at concentration equal to IC_50_, increased to 0.47 and was significantly higher than M2 values estimated for BJ (0.17 and 0.33) and HaCaT (0.10 and 0.33), respectively. This points to the possible action of G3-BCLF within mitochondria of glioblastoma cells. This is particularly important since recently, mitochondria has become a promising target of anticancer therapy. It has been confirmed that cancer transformation is accompanied by the deregulation and disruption of many crucial mitochondrial functions. Recognized hallmarks of cancer such as uncontrolled proliferation, disabled apoptosis, acquired migration (invasiveness) and primarily switch in energy metabolism from ox-phosph to glycolytic pathway depend on deregulations of respective mitochondrial signal transduction pathways (for a review see [74]). It has been shown that celecoxib and PPARγ agonists exert influence on the mitochondria-dependent apoptosis and metabolism [75,76]. The high cytotoxic effect of G3-BCL in glioma and BJ cells may be explained by the observation that it was accumulated at a higher level in mitochondria of these cells as compared with HaCaT mitochondria (Figure 4).

### 2.5. Intracellular ATP Level

**Figure 5:**
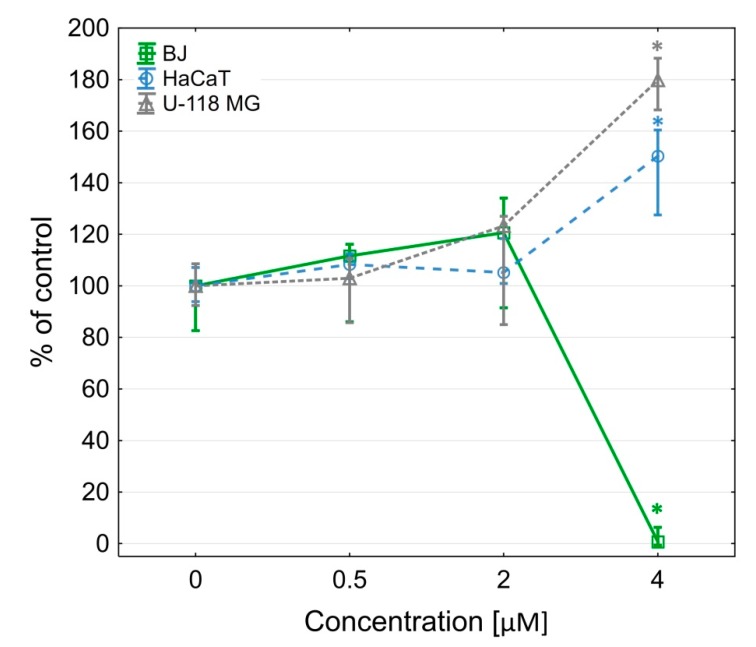
Intracellular ATP level in BJ, HaCaT and U-118 MG cells after 24 h treatment with G3-BCL. Results are presented as median of triplicates from three independent experiments and expressed as % of non-treated controls. The whiskers are lower (25%) and upper (75%) quartile ranges. * P < 0.05 (Kruskal–Wallis test).

Estimation of cellular ATP level revealed that PAMAM G3-BCL conjugate at 0.5–2µM concentrations did not significantly affect the level of nucleotide, and the energy homeostasis remained stable in all investigated cell lines. However, at cytotoxic concentrations higher than 4 µM, BJ cells were depleted of ATP and dead, whereas in both HaCaT and glioma cells, a significant increase of ATP level was observed of up to 150% and 180% of the control, respectively (Figure 5). This generation of energy in glioma cells may be explained by the effective switch from OX-PHOS mechanisms to the glycolytic pathway characteristic of cancer cells [74].

### 2.6. Apoptosis and Late Apoptosis/Necrosis

**Figure 6:**
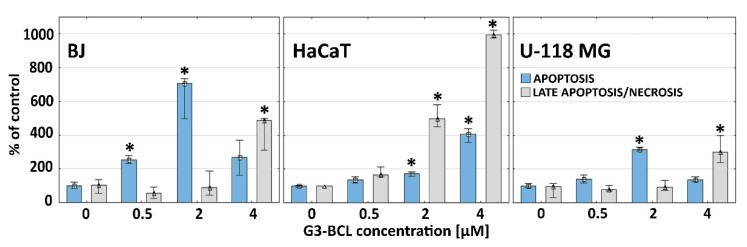
Caspase 3/7 activity and late apoptotic/necrotic cell population in BJ, HaCaT, and U-118 MG cells after 24 h treatment with G3-BCL conjugate. Results are medians of triplicate from three independent experiments expressed as % of the non-treated controls. The whiskers are lower (25%) and upper (75%) quartile ranges. * P < 0.05 (Kruskal–Wallis test).

A very different profile of caspase 3/7 activity as marker of apoptosis (A) [77] and penetration of 7-ADD into cells as a marker of late apoptotic/necrotic (LA/N) phase [78] were seen in the investigated cell lines. It has been established that intracellular levels of ATP, supplied through glycolysis or oxidative phosphorylation, determines cell death by apoptosis, favored by a high level of intracellular ATP or necrosis, promoted by low energy level [79,80]. Fibroblasts enter the apoptotic pathway measured as caspases 3/7 activity at non-toxic 0.5 µM G3-BCL concentration with significant increase up to 2 µM concentration (in the range of IC_50_ value) with constant ATP supply. At 4 µM conjugate, a significant increase of LA/N was observed that correlates with depleted cellular ATP level (Figure 6) [81]. It has been shown by others that in fibroblasts, the effector caspase activation caused poly(ADP-ribose)polymerase-1 (PARP-1) cleavage with maintained ATP level and induction of apoptosis [82]. Similarly, in glioma cells, the increase of caspases 3/7 activity and induction of apoptosis was dominant up to 2 µM (in range of IC_50_) concentration, whereas the change to LA/N processes was seen at 4 µM concentration despite of high cellular ATP level. However, those markers were at a much lower level than in fibroblasts (Figure 6). The propensity of astrocytic glioma to necrosis was described by Furnari et al. [83].

In HaCaT cells, which were most resistant to G3-BCL cytotoxicity, a higher participation of LA/N was seen from 2 µM concentration as compared to low level of apoptotic marker, caspases 3/7 activity, despite of high ATP level. Similar observations were obtained by Nzengue, et al. [84] who investigated the mechanism of cell death in HaCaT keratinocytes and rat glioma C6 cells. The resistance of HaCaT cells to apoptosis was also described by other authors, who have shown that many effectors which induce apoptosis in other cell types did not affect HaCaT keratinocytes. Of the tested stimuli including interferon gamma, tumor necrosis factor alpha, interleukin-4 and muramyl dipeptide, only interferon gamma induced HaCaT cell apoptosis [85,86]. This may be explained by spontaneous mutations in both alleles of pro-apoptotic protein p53 in immortalized HaCaT cells that affect the apoptosis pathway [87,88] and may also increase this cell line resistance to the G3-BCL construct.

Similar viability and apoptotic/necrotic response of BJ and glioma cells to conjugate treatment and a different pattern of HaCaT response indicate that composed action of both drugs on induction of cell death mechanisms was independent on p53 status. It has been shown that celecoxib alone caused no significant difference in apoptosis level independent of p53 status of various tested glioma cell lines [32].

### 2.7. Effect of G3-BCL on Cell Migration

**Figure 7:**
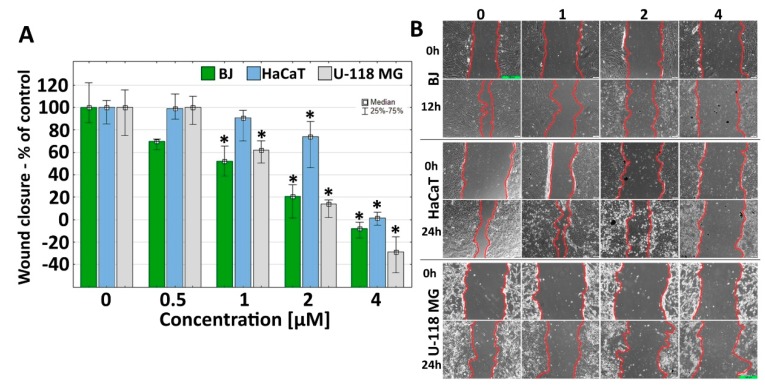
(**A**) G3-BCL effect on BJ, HaCaT, and U-118 MG cell migration after 12 and 24 h treatment. Results are presented as median of triplicate from three independent experiments and expressed as % of non-treated controls. The whiskers are lower (25%) and upper (75%) quartile ranges. * P < 0.05; (Kruskal–Wallis test). (**B**) Images from contrast-phase inverted microscope. Scale bar equal to 300 µm.

G3-BCL conjugate at concentrations in the range below IC_50_ (1 µM) decreased GBM and human fibroblast cells mobility significantly (to 45% and 40% of controls, respectively) and a decrease of migration rate at 2 µM concentration was seen in HaCaT cells (to 70% of control) (Figure 7).

The anti-migration property of G3-BCL was visible only at cytotoxic concentrations estimated with an XTT assay (Figure 1). At 2–4 μM, dendrimer concentration for BJ and HaCaT cells, and 1–4 μM for U-118 MG, inhibition of cells migration was caused by toxic and anti-proliferative effect of the tested compound. This was also confirmed by apoptosis and late apoptosis/necrosis occurrence at the mentioned dendrimer concentrations. Anti-migratory activity of celecoxib was observed in various cancer cell lines at clinically achievable concentrations (2.5–5.0 μM) [89,90,91,92]. In human glioblastoma SHG-44 cells the anti-metastatic effect of celecoxib was observed at 30–150 µM concentration [93]. Parecoxib, the prodrug of valdecoxib the COX-2 antagonist from the same group as celecoxib, inhibits in vitro GBM cell proliferation, migration and invasion at concentrations of 50–100 μM [64]. In addition, increasing evidence suggests that PPARγ agonists, including thiazolidinediones and nonthiazolidinediones, inhibit the motility and invasiveness of glioma cells [94]. Very invasive behavior of GBM is characteristic for that cancer [3,95], hence the observed anti migratory effect of G3-BCL conjugate at much lower concentration than either drug alone may efficiently prevent or limit of the GBM penetration into surrounding tissues.

### 2.8. COX-2 Expression and PGE_2_ Production

**Figure 8:**
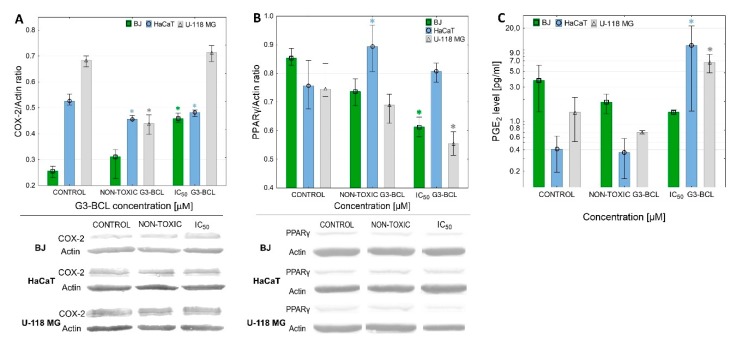
Effect of G3-BCL on expression of COX-2 protein (**A**), PPARγ (**B**) and PGE_2_ production (**C**) in BJ, HaCaT and U-118 MG cells after 24 h treatment with non-toxic (0.5 µM) or IC_50_ concentrations (estimated with XTT assay). Levels of COX-2 protein were calculated against β-actin and expressed as the percent of the control. Squares indicates medians and whiskers corresponding to the lower (25%) and upper (75%) quartile ranges. * P < 0.05 significant difference against control (Kruskal–Wallis test).

All investigated cell lines exhibited a basal level of COX-2 protein expression and production of PGE_2_ (Figure 8). It has been confirmed that in fibroblasts, the level of COX-2 protein was very low and significantly higher levels were seen in U-118 MG cells. In addition, non-stimulated HaCaT cells presented higher levels of COX-2 as compared to normal BJ cells (Figure 8). The observation that high COX-2 expression correlated with glioma histological grade but not with positive p53 immunostanning was made by Shono et al. [20].

The G3-BCL conjugate at non-cytotoxic and at IC_50_ concentrations induced a conventional response in BJ cells with a significant increase of COX-2 expression, without effecting PGE_2_ production. In U-118 MG cells, a concentration-dependent effect on COX-2 protein level was observed; inhibition at low, non-cytotoxic and up-regulation at IC_50_ concentration was accompanied with an increase of PGE_2_ production rate. In HaCaT cells G3-BCL induced a low but significant decrease of COX-2 protein expression, although production of PGE_2_ was significantly higher at IC_50_ concentration of G3-BCL, which may be the result of COX-1 activity. It has been noticed that in HaCaT cells the COX-2 inhibitor did not fully block PGE_2_ release [96].

Basal levels of PPARγ protein were not significantly different in all three cell lines. The effect of G3-BCL conjugate on PPARγ expression was similar in BJ and U-118 MG cells and resulted in a decrease of the expression of PPARγ protein at IC_50_ concentrations of conjugate without significant change in HaCaTs. Exposition of various primary human lung tumor cells to celecoxib (IC_50_ values between 12 and 43 μM) resulted with apoptosis and upregulation of PPARγ and COX-2 at mRNA and protein levels [97,98]. Our results demonstrated that conjugate with both celecoxib and Fmoc-L-Leucine caused apoptosis of U-118 MG cells at much lower concentration (IC_50_ = 1µM) with significant decrease of the expression of PPARγ. At non-cytotoxic concentration, G3-BCL stimulated U-118 MG proliferation to 120% of control value with parallel significant decrease of COX-2 expression, followed by its upregulation at IC_50_ concentration with significant increase of PGE_2_ production and decrease of proliferation rate. That allowed us to conclude that the pro-apoptotic and anti-migratory effect of G3-BCL conjugate was realized by other mechanisms than the influence of PPARγ expression but it was dependent on COX-2 protein level and high PGE_2_ production. Comparison of the G3-BCL effect on BJ fibroblasts, having active p53 signaling pathway with p53 mutated U-118 MG cell line, revealed that regardless of p53 status, the significant pro-apoptotic effect was observed. Similar observations were made by Kang et al. with various phenotypes of glioma cell lines [32]. Also, the very different response of HaCaT cells to G3-BCL confirmed that mutated p53 may result in various sensitivity to effectors of COX-2 and PPAR-γ activity.

## 3. Materials and Methods

### 3.1. Dendrimers

Third generation biotinylated PAMAM G3 dendrimer conjugated with 16 celecoxib molecules and 15 Fmoc-L-Leucine molecules (G3-BCL) and its fluorescein molecule labeled analog (G3-BCLF) were synthetized as described earlier [39].

### 3.2. Materials

Eagle’s Minimum Essential Medium (EMEM), Dulbecco’s Modified Eagle’s Medium (DMEM), fetal bovine serum (FBS), penicillin and streptomycin solution were purchased from ATCC (Manassas, VA, USA). Trypsin-EDTA solution, phosphate-buffered saline (PBS) with and without magnesium and calcium ions, 0.4% trypan blue solution, fluorescent marker DAPI (4′,6-diamidino-2-phenylindole, dihydrochloride), 7-AAD, LysoTracker Red, MitoTracker Deep Red FM, CyQUANT^®^GR Cell Proliferation Assay Kit were purchased from Thermo Fischer Scientific (Waltham, Massachusetts, USA). XTT sodium salt (2,3-bis [2-methoxy-4-nitro-5-sulfophenyl]-2Htetrazolium-5-carboxanilide inner salt), phenazinemethosulfate(PMS), N-methyl dibenzopyrazine methyl sulfate were purchased from Sigma-Aldrich (St Louis, MO, USA). Rabbit anti PPARγ antibody purchased from WuhanFine Chemicals Co. Ltd. (Wuhan, China), rabbit anti-COX-2 polyclonal antibody from Novus Biologicals (Centennial, Colorado, USA) and goat anti-rabbit IgG (H+L) antibody from Jackson ImmunoResearch (Philadelphia, PA, USA). ELISA Prostaglandin E2 Parameter Assay Kit was delivered by R&D Systems (Minneapolis, Minnesota, USA) and DAB reagent by EMD Millipore (Burlington, Massachusetts, USA). Cell culture dishes were from Corning Incorporated (Corning, NY, USA), Greiner (Kremsmünster, Austria) or Nunc (Rochester, New York, USA).

### 3.3. Cell cultures

Human immortalized keratinocytes (HaCaT) obtained from Cell Lines Service (Eppelheim, Germany) and human glioblastoma cell line (U-118 MG) obtained from ATCC (Manassas, VA, USA) were cultured in DMEM (doubling time 24 and 35 h, respectively). Normal fibroblasts BJ purchased from ATCC (doubling time 1.9 days) were grown in EMEM medium. Media were supplemented with 10% heat-inactivated FBS and 100 U/mL penicillin, and 100 μg/mL streptomycin. Cells were incubated at 37 °C in humidified 95% air with 5% CO_2_. Media were changed every 2–3 days and cells passaging at 70–80% confluence after treatment with 0.25% trypsin-EDTA/PBS (calcium and magnesium free). Cell morphology was checked using a Nikon TE2000S Inverted Microscope (Tokyo, Japan) with phase contrast. Number and viability of cells were estimated by trypan blue exclusion test using Automatic Cell Counter TC20TM (Bio-Rad Laboratories, Hercules, CA, USA). All assays were performed in triplicate from three independent experiments.

#### 3.3.1. Cytotoxicity

Cytotoxicity of the G3-BCL conjugate was determined with XTT assay that estimate the capacity of mitochondrial oxidoreductases to reduce XTT into water-soluble, formazan product [99]. Cells were seeded in flat-bottom 96-well plates at density 1 × 10^4^ cells/well (BJ and U-118 MG cells) or 2 × 10^4^ (HaCaT cells) and allowed to grow for 24 h. Working solutions of dendrimers (0.5–4 µM) were prepared in culture media. The DMSO concentration was adjusted to 0.1% in all samples that had no significant effect on treated cell lines. After 24 h exposure to dendrimers, the medium was removed, XTT mixture of 1.7 mM of XTT and 8.3 μM of PMS in the complete medium was added (100 µL/well) and the plates were returned to the incubator for 1 h. Then absorbance was measured at 450 and 620 nm against a blank sample (100 μL of complete growth medium containing XTT and PMS), using a microplate reader (μQuant – BioTek, Winooski, VT, USA).

#### 3.3.2. Proliferation

Cell proliferation after treatment with G3-BCL was determined by CyQUANT^®^GR Cell Proliferation Assay Kit, a fast and sensitive tool for counting cells in a microplate format. This kit is based on measurement of cellular DNA content using fluorescent dye that enhances its fluorescence after binding to cellular nucleic acids. The DNA content is proportional to the number of cells. Cells were seeded in 96-well clear plates 5 × 10^3^ cells/well. After 24 h, the incubation medium was removed, replaced with a G3-BCL dendrimer at concentrations ranging from 0.125 to 4 μM (200 μL/well) and incubated 72 h. Assay was performed according to manufacturer protocol. The fluorescent signal, proportional to the number of cells, was measured with Tecan Infinite M200 PRO Mulitmode Microplate Reader (TECAN Group Ltd., Männedorf, Switzerland) at 360/460 nm. The results were expressed as percentage of the non-treated control.

#### 3.3.3. Cellular Uptake of G3-BCLF

Cells were grown in 96-well plates at 2 × 10^4^ cells/well (BJ and U-118 MG) or 4 × 10^4^ (HaCaT). After 24 h, cells were washed with PBS and treated with FITC labeled analog of G3-BCL (G3-BCLF) at 0.1 µM concentration in complete EMEM medium for 1, 3, 6 or 24 h. After incubation cells were fixed in 3.7% formaldehyde and washed with PBS. 600 nM DAPI in PBS was added (100 µL/well) for 1 h at RT. Fluorescence signal was monitored at 485/530 nm for FITC or 360/460 nm for DAPI using Infinite M200 PRO Multimode Microplate Reader (TECAN Group Ltd., Switzerland). The DAPI fluorescence signals were used to estimate number of cells in each well and to calculate fluorescent signal intensity per cell using the constructed calibration curve.

#### 3.3.4. Intracellular Location of G3-BCLF

Cells were seeded in Nunc™ Lab-Tek™ 8-well Chambered Coverglass at density 6 × 10^4^ (BJ and U-118 MG) or 1.2 × 10^5^ (HaCaT) in 400µL medium/well. After 48 h, incubation medium was replaced with non-toxic (0.5 μM) or equal to IC_50_ concentrations of G3-BCLF as estimated earlier (IC_50_=1.25, 1.29, and 4.49 µM for U-118 MG, BJ and HaCaT, respectively). After 24 h, lysosomes were labelled with 100 nm solution of LysoTracker Red and mitochondria in parallel samples with 100 nm MitoTracker Deep Red FM solution in medium for 30 min at 37 °C. Washing, fixation with 3.7% formaldehyde and labeling nuclei with 600 nm DAPI solution were performed as described above. Images were collected using Olympus FV10i confocal microscope with 60× objective with water immersion (total magnification 120× ). Fluorescence signal was recorded at 405/461 nm for DAPI, 485/535 nm for FITC or 559/600 nm for Lyso or MitoTracker. Pinhole was set for 1 AU (airy unit) and obtained images had an optical section thickness of approximately 1.02 μm. Images were analyzed using ImageJ software with JACoP plugin and Mander’s colocalization coefficients were estimated.

#### 3.3.5. Cellular ATP Level

Cells were seeded in 96-well plates at 2 × 10^4^ cells/well for HaCaT and 1 × 10^4^ for U-118 MG and BJ and incubated for 24 h. Solution of G3-BCL in media with FBS was added at concentration range 0.5–4 μM (100 μL/well). After 24 h. CellTiter-Glo® reagent was added into each well and assay was performed according to manufacturer’s protocol. Luminescence was measured with Tecan Infinite M200 PRO Multimode Microplate Reader (Tecan Group Ltd., Switzerland). The DAPI fluorescence signals were used to estimate number of cells in each well and to calculate luminescent signal intensity per one cell from calibration curve. Results were expressed as % of non-treated control.

#### 3.3.6. Apoptosis and Late Apoptosis/Necrosis

To assess active apoptotic (A) process, caspase 3 and 7 activity measurement was performed using the Apo-ONE® Homogenous Caspase-3/7 Assay (Promega, Madison, WI, USA) based on caspase ability to cleave the substrate that emits green fluorescence. Cells were seeded in black 96-well, clear bottom plates and treated as described for ATP assay. After 24 h assay was performed according to manufacturer’s protocol. After incubation (120 min, RT) fluorescence was measured at 490/530 nm using Infinite M200 PRO Multimode Microplate Reader. Late apoptosis/necrosis (LA/N) evaluation was performed with 7-amino-actinomycin D dye (7-AAD) [78]. Cells were seeded in a black 96-well, clear bottom plate at 4 × 10^4^ cells/well for HaCaT and 2 × 10^4^ for U-118 MG and BJ cells and treated as described above for caspase assay. 7-AAD (0.01 mg/mL in PBS) was added. and after incubation for 30 min at 37 °C labeling of nuclei with DAPI was performed as described above. Fluorescence intensity for 7-AAD (546/647 nm) and DAPI (360/460 nm) was measured with Infinite M200 PRO Multimode Microplate Reader. Results were presented as 7-AAD/DAPI signal ratio that corresponds to the percent of LA/N cells in the whole cell population.

#### 3.3.7. Cell Mobility

BJ, HaCaT and U-118 cells were seeded into 24-well plates at 1 × 10^5^, 2 × 10^5^ and 2 × 10^5^ cells/well, respectively. Anti-migration properties of G3-BCL were estimated with wound healing assay as described earlier [100].

#### 3.3.8. COX-2 and PPARγ Expression and PGE_2_ Production

After 24 h treatment with dendrimers (0.5 µM non-toxic and IC_50_ concentrations estimated with XTT assay), proteins from cell cultures were isolated, separated by SDS/PAGE and transferred into PVDF membrane (0.2 μm, Bio-Rad, Hercules, CA, USA) as described [39]. Membranes were blocked with 1% bovine serum albumin (BSA) in TBS/T for 2 h. All incubations were done on a rotary shaker at RT. The membranes were washed once (10 min) in TBS containing 0.05% (*v*/*v*) Tween 20. Rabbit anti-PPARγ antibody (WuhanFine Chemicals Co. Ltd., Wuhan, China) and rabbit anti-COX-2 polyclonal antibody (Novus Biologicals) were diluted in 1% BSA-TBS/T, 1:4000 and 1:2000 respectively. The membranes were incubated with primary antibodies for 2 h. After four times washing (5 min) in TBS/T, secondary peroxidase conjugated antibody (goat anti-rabbit IgG (H + L) diluted 1:10,000 in TBS; (Jackson ImmunoResearch Laboratories, Baltimore, PA, USA) was added. After 1 h, membranes were washed two times (5 min) in TBT/T, two times (5 min) in TBS, developed with DAB reagent (10 min, RT) and collected with Epson SX235 scanner. Results were quantified with ImageJ software (US National Institute of Health, Bethesda, MD, USA).

To determine PGE_2_ level cells were cultured as described in a motility assay. After 24 h treatment with G3-BCL culture medium was collected and ELISA Prostaglandin E2 Parameter Assay Kit (R&D Systems) was performed in triplicate according to manufacturer protocol.

#### 3.3.9. Statistical Analysis

To estimate differences between G3-BCL treated and non-treated samples the non-parametric Kruskal–Wallis test was used. Mann-Whitney U test was performed to estimate differences in amount of G3-BCLF in cellular compartments at non-toxic or IC_50_ concentrations. P < 0.05 was considered as statistically significant. Calculations were performed with Statistica 12.5 software (StatSoft, Tulsa, OK, USA).

## 4. Conclusions

Comparison of the effect of G3-BCL on expression of COX-2 and PPARγ protein and PGE_2_ production of three different cell line phenotypes reveal that anti-proliferative, pro-apoptotic and anti-migratory effects of the conjugate was realized by other mechanisms than an influence on PPARγ expression regardless of p53 cell status, but it was dependent on COX-2 protein level and high PGE_2_ production.

The important achievement was the observation that G3-BCL significantly decreased viability, proliferation and mobility of U-118 MG cells at concentration in range of 1–2 µM, many fold lower than either drug applied alone. Thus, G3-BCL might be a potential candidate for the targeted, local glioma therapy with limited side effects.

The obtained results also shed light on the use of HaCaT cell line as model for toxicity tests. Many authors emphasize this possibility, but others do not. Our experiments revealed that the profile of HaCaT response to G3-BCL conjugate differs significantly from normal fibroblasts and U-118 MG glioma cells, which makes them more resistant to the investigated compounds. That confirms the limitation of HaCaT cells as a model for evaluation of drug toxicity.
